# Analytical Methods for Anatoxin-a Determination: A Review

**DOI:** 10.3390/toxins16040198

**Published:** 2024-04-19

**Authors:** Cristina Plata-Calzado, Ana I. Prieto, Ana M. Cameán, Angeles Jos

**Affiliations:** Area of Toxicology, Faculty of Pharmacy, Universidad de Sevilla, Profesor García González 2, 41012 Seville, Spain; cpcalzado@us.es (C.P.-C.); camean@us.es (A.M.C.); angelesjos@us.es (A.J.)

**Keywords:** anatoxin-a, analytical methods, cyanotoxins, parameter validation

## Abstract

Anatoxin-a (ATX-a) is a potent neurotoxin produced by several species of cyanobacteria whose exposure can have direct consequences, including neurological disorders and death. The increasing prevalence of harmful cyanobacterial blooms makes the detection and reliable assessment of ATX-a levels essential to prevent the risk associated with public health. Therefore, the aim of this review is to compile the analytical methods developed to date for the detection and quantification of ATX-a levels alone and in mixtures with other cyanotoxins and their suitability. A classification of the analytical methods available is fundamental to make an appropriate choice according to the type of sample, the equipment available, and the required sensitivity and specificity for each specific purpose. The most widely used detection technique for the quantification of this toxin is liquid chromatography–tandem mass spectrometry (LC-MS/MS). The analytical methods reviewed herein focus mainly on water and cyanobacterial samples, so the need for validated analytical methods in more complex matrices (vegetables and fish) for the determination of ATX-a to assess dietary exposure to this toxin is evidenced. There is currently a trend towards the validation of multitoxin methods as opposed to single-ATX-a determination methods, which corresponds to the real situation of cyanotoxins’ confluence in nature.

## 1. Introduction

**Figure 1:**
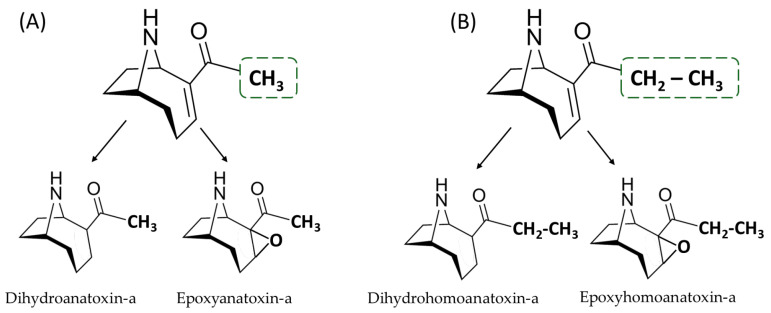
Structures of (**A**) ATX-a and (**B**) HATX-a and their respective derivatives.

Anatoxin-a (ATX-a) belongs to the group of cyanobacterial toxins, which includes other well-known members such as microcystins (MCs) and cylindrospermopsin (CYN). Chemically, it is a bicyclic amine alkaloid whose molecular formula is C_10_H_15_NO. Its molecular weight is 165.23 g/mol, and it is very soluble in water. ATX-a, its analog homoanatoxin-a (HATX-a), and its dihydro derivatives (Figure 1) are produced by different cyanobacterial genera, including *Anabaena*, *Dolichospermum*, *Aphanizomenon*, and *Cuspidothrix*, among others, with a worldwide distribution. However, ATX-a has generally been detected less frequently than MCs and CYN [1]. This could be related to its degradation through photolysis and non-photochemical reactions under natural conditions [2,3]. In any case, ATX-a also has a significant occurrence, and, for example, Bouma-Gregson et al. [4] found ATX-a in 58.9% of the benthic mat samples they analyzed, while Moreira et al. [5] found that 33% of the samples from seven freshwater ecosystems in Portugal were above the guideline value established for ATX-a in Portuguese freshwater systems. Moreover, this toxin has been detected jointly with other cyanotoxins such as MCs [6,7] or CYN [8]. In certain cases, ATX-a has even been detected at higher levels than MCs, as is the case for samples collected from the Eel River (California), alerting us to the importance of this toxin [4].

The growing occurrence and bioaccumulation of cyanotoxins [9,10] raises concerns about their potential risks. These risks are based on two main factors: hazards and exposure. Regarding the hazards, ATX-a is a well-known neurotoxin. It is a nicotinic agonist and has been shown to bind to acetylcholine receptors and cause the inhibition of the enzyme acetylcholinesterase. Consequently, the neurons continuously propagates neuronal impulses that lead to nerve depolarization through the movement of positively charged ions across the receptor, in addition to the desensitivity of the receptor, blocking neurotransmission [11,12,13]. However, toxicity data for ATX-a are comparatively scarcer than for other cyanotoxins and are a topic worthy of research. Recently, Plata-Calzado et al. [14] reviewed the toxic effects produced by ATX-a under laboratory conditions and evidenced that, in addition to neurotoxic effects, the scientific literature shows that ATX-a can also have immunotoxic effects [15,16] and that oxidative stress and apoptosis are the mechanisms involved. Moreover, ATX-a was shown to be genotoxic in vitro by the micronucleus test [17].

With regard to field studies and case reports, ATX-a has been involved in plenty of animal intoxication cases, which has led to great concern [3,18]. In most of the detected cases, ATX-a caused significant behavioral changes, vomiting, ataxia, locomotor deficiency, respiratory distress, and even death in different animal species, such as ducks, cows, flamingos, etc., with dogs being the animal in which most cases of poisoning have been detected [3]. Concentrations of up to 8700 μg/L ATX-a have been detected in their stomach contents [19], and concentrations up to 357 mg/kg have been detected in dogs’ vomit [20].

In contrast, human data poisonings specifically attributed to ATX-a are not so evident in the literature, as co-exposure with other additional cyanotoxins has been described [1]. However, Biré et al. [21] reported human poisoning in 26 patients due to the consumption of sea figs in France between January 2011 and March 2018. These patients presented with diarrhea, nausea, vomiting, paresthesia, dizziness, and headache, among other symptoms that evoke a cerebellar syndrome. This study reported ATX-a concentrations ranging from 193.7 to 1240.2 µg/kg in sea figs (*Microcosmus*), lower than those reported to cause toxic effects in animals [1]. This emphasizes the toxicological relevance of ATX-a in humans. Nevertheless, the database of ATX-a toxicity is still limited, and that is the reason why the World Health Organization (WHO) has not yet established a long-term provisional health-based reference value [1].

With respect to the exposure data for ATX-a, it has already been stated that it has a worldwide distribution. The levels reported in the waters are very variable. In open water, the concentrations rarely exceed 10 of µg/L, but they can exceed 1000 µg/L in surface blooms, and in drinking water concentrations, they vary from the low-µg/L range to 8.5 µg/L [1]. Also, in relation to water, recreational activities can be another form of exposure, either by ingestion or inhalation, though this has a lower impact. Regarding oral exposure, the ingestion of contaminated food is another possibility to take into account. Currently, data on the occurrence of ATX-a in food are still limited. However, concentrations of up to 33.00 μg/g of this toxin have been detected in dietary supplements containing cyanobacteria [22]. In relation to seafood, in addition to the study by Biré et al. [21] mentioned above, Amzil et al. [23] detected, for the first time, the presence of this neurotoxin in mussels in France. Moreover, interestingly, different studies have investigated the potential bioaccumulation of ATX-a in fish. Osswald et al. [24] calculated a bioaccumulation factor of 2.65 in juvenile *Cyprinus carpio* exposed to cells of ATX-a producing strains of *Anabaena* sp. Osswald et al. [25] determined a bioconcentration factor ranging from 30 to 47 based on fresh weight in juvenile *Oncorhynchus mykiss* exposed to ATX-a. Thus, they concluded that it was necessary to consider potential human exposure through the ingestion of contaminated fish. Similarly, Pawlik-Skowrońska et al. [26] reported the simultaneous accumulation of ATX-a (up to 30 ng/g fresh weight, muscle) and MCs in three species of fish. On the contrary, Colas et al. [27] did not observe ATX-a accumulation in medaka fish.

For all of this, the exposure assessment must rely on analytical results obtained by techniques and validated methods that allow for the unequivocal determination and quantification of ATX-a. In this sense, there are plenty of analytical methods for ATX-a detection that have been noted in the scientific literature. Thus, the aim of this work was to review the research papers focused on analyzing ATX-a, both alone and in combination with other cyanotoxins, and its classification to facilitate the selection of an appropriate method based on the type of sample (matrix), the available equipment, and the required sensitivity and specificity for each specific purpose. Previously, other reviews have been published looking at methods for the determination of cyanotoxins, including ATX-a [28,29]. However, these works mainly focus on extraction procedures prior to sample analysis and do not consider the validation parameters of the methods, which are important when selecting the most appropriate technique according to the requirements of each specific scenario (type of matrix, toxin recoveries, LOQ, LOD, etc.). Furthermore, they do not focus on ATX-a analytical methods, so these studies do not cover all of the existing literature on ATX-a analytical methods. For all this, the novelty of the present review lies in the fact that it summarizes and classifies all the validation parameters of each method according to the type of sample and detection system used and provides a complete overview and update of all available information up to 2023, which allows one to obtain a picture of the benefits and drawbacks of each method. This review aims to support those who need to work with ATX-a for research, surveillance, or monitoring purposes in their decision-making process to select the best analytical option available currently. Also, it will contribute to improving ATX-a exposure assessments in the framework of risk evaluations.

## 2. Analytical Methods for ATX-a Determination

**Figure 2:**
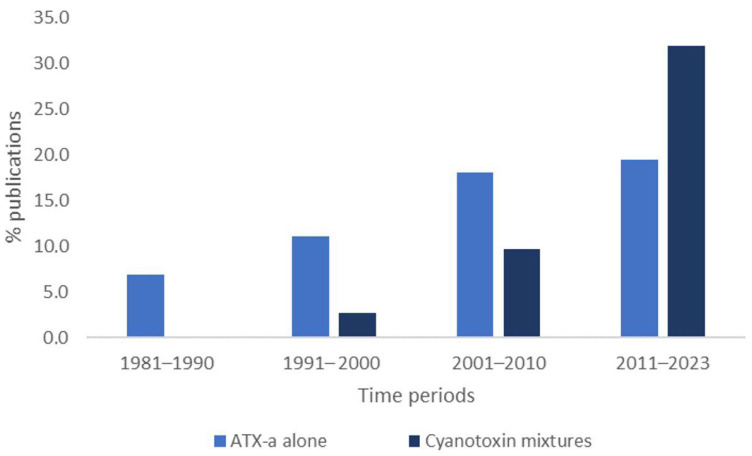
% of publications focused on determination of ATX-a alone or in combination with other cyanotoxins in relation to years of publication.

The first studies focused on the validation of methods for the determination of ATX- date back to the 1980s, focusing only on ATX-a (Figure 2). However, the trend in recent years has shifted towards the development of methods that allow for the detection of several cyanotoxins simultaneously, as this is a more common scenario in nature. In this case, with regard to multitoxin methods, only those methods for the determination of cyanotoxin mixtures containing ATX-a will be discussed. In addition, the need for the development of rapid and reliable methods considering both scenarios (ATX-a alone or in combination) has led to further research into new methodologies.

**Figure 3:**
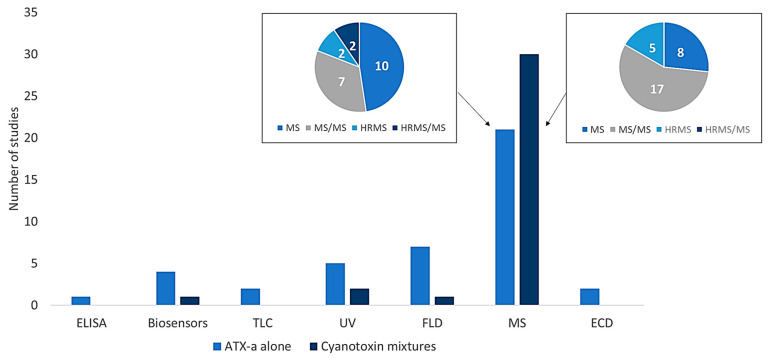
Techniques employed for determination of ATX-a alone or in combination with other cyanotoxins. ELISA: enzyme-linked immunosorbent assay; TLC: thin-layer chromatography; UV: ultraviolet; FLD: fluorescence detection; MS: mass spectrometry; ECD: electron capture detector; MS/MS: tandem mass spectrometry; HRMS: high-resolution mass spectrometry: HRMS/MS: high-resolution mass spectrometry tandem mass spectrometry.

The methods developed for ATX-a analysis employ a variety of detection systems. One of the first techniques used for the isolation and purification of ATX-a was thin-layer chromatography (TLC), which is, in addition, a low-cost and swift screening technique [30,31]. However, it is a very basic technique. Therefore, more complex techniques have been developed, such as gas or liquid chromatography coupled with spectroscopy or mass spectrometry (MS) detection. Additionally, immunochemical assays or biosensors can also be employed for its detection (Figure 3). However, more than half of the methods have been performed with MS detection systems, highlighting the tandem mass system (MS/MS) mainly for the analysis of cyanotoxin mixtures.

**Figure 4:**
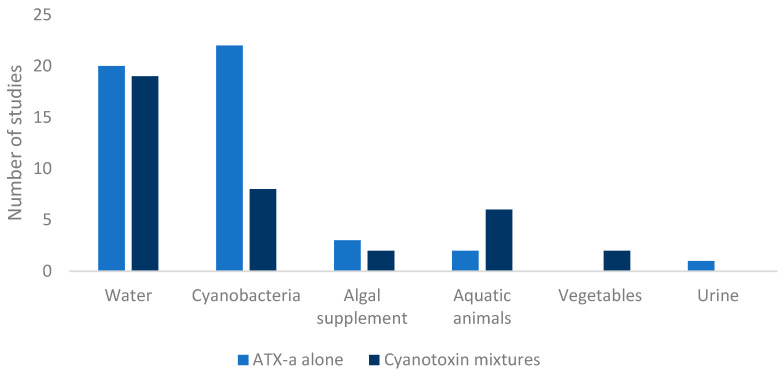
Matrices of the samples employed for the determination of ATX-a alone or in combination with other cyanotoxins.

Based on the type of sample analyzed in the revised articles, most of the methods developed for the determination of ATX-a are in water (54.2%) and cyanobacterial cultures (41.7%), compared to methods in which the presence of ATX-a is analyzed in more complex matrices such as aquatic animals (11.1%), vegetables (2.8%), or algae-based supplements (6.9%) (Figure 4).

To our knowledge, all the methods for the determination of ATX-a alone are listed in Table 1 and Table S1 (Supplementary Materials). In addition, multitoxin methods that include ATX-a determination are summarized in Table 2 and Table S2 (Supplementary Materials).

### 2.1. Enzyme-Linked Immunosorbent Assay (ELISA)

Cyanotoxins can be determined and quantified by their interactions with specialized antibodies, commonly used to determine other cyanotoxins such as MCs, saxitoxins (STXs), and nodularins (NODs). In fact, in 2016, the US Environmental Protection Agency (US EPA) validated an immunoassay for the determination of MCs and NODs [84]. On the contrary, specific antibodies against ATX-a were not available until recently. For the first time, Cevallos-Cedeño et al. [54] developed a sensitive immunoassay for ATX-a after the same research group generated immunoreagents (bioconjugates and antibodies) following the synthesis of three different functionalized derivatives of ATX-a [85], allowing ATX-a to be controlled at lower part-per-billion levels. The authors also used these immunoreagents to optimize a dipstick assay that provides rapid screening in water samples with levels higher than 2 ng/mL ATX-a [54]. This advancement has made the determination of this cyanotoxin possible through commercial kits (Abraxis, Warminster, PA, USA). In this regard, the kit based on the recognition of toxins by a monoclonal antibody shows a limit of detection (LOD) of around 0.1 µg/L, with recoveries between 98 and 104.4%. However, it is important to keep in mind the limitations of this rapid screening method. This test may experience interferences in more complex matrices; the presence of solvents should be reduced to avoid the matrix effect (methanol concentration should be <2.5%.), and in those cases where positive results require regulatory measures, it is also necessary to perform an analysis using an alternative method.

### 2.2. DNA Aptamers/Biosensors

Aptamers are single-stranded DNA or RNA sequences that take the form of unique three-dimensional structures, enabling them to recognize a specific target with high affinity. These have multiple applications, including biosensors, which are analytical instruments that allow for the detection of an analyte of interest by means of biorecognition and result in a measurable signal. In this respect, biosensors using ATX-a specific DNA aptamers have been published using different detection modes, such as impedimetric [45], electrical conductance [49], colorimetric [51], and electrochemiluminescence [52] aptasensors. The DNA aptamer characterized by Elshafey et al. [45] was one of the first to be developed. The technique revealed an LOD of 0.5 nM, a linear range for ATX-a concentrations ranging from 1 to 100 nM, and recoveries between 94.8 and 108.6%. In general, these methods have lower LODs than other techniques, even detecting ATX-a at picomolar levels [51]. In addition, aptamers exhibit many advantages because they are simple, specific, and cost-effective, as they do not require complex instrumentation, which makes them a useful tool for field studies and allows for the analysis of a large number of samples in a short time, which is a suitable screening method [86]. However, only Li et al. [73] have developed a fluorescence sensor-based adaptor method for the detection of cyanotoxin mixtures, including ATX-a, CYN, NOD, and MC-LR, in water samples. This method shows very good recoveries (in the range of 97.0–104.6%), proving to be a simple, sensitive, and robust method for the determination of four of the most common cyanotoxins in field samples.

### 2.3. Ultraviolet and Fluorescence Detection

The early methods developed based on ATX-a determination mainly employed high-performance liquid chromatography (HPLC) coupled with absorbance detection techniques such as ultraviolet–visible (UV-vis) or fluorescence spectroscopy, providing both qualitative and quantitative data in ATX-a analyses. However, most of these methods contained little detailed data on the validation parameters [30,87,88,89,90,91]. Due to the presence of an α, β-unsaturated ketone, ATX-a has maximum UV absorption at 227 nm, allowing for its discrimination from phenylalanine (257 nm), a compound that frequently produces interferences with ATX-a [92]. For UV detectors, the LOD to determine ATX-a can be up to 0.025 µg/L in water samples [91]. Nevertheless, despite being a fast and simple method, it has limitations, such as its low sensitivity and the numerous interferences in more complex matrices, such as environmental samples or animal and plant tissues, causing false positives or negatives. In addition, HPLC-UV does not allow for the detection of ATX-a degradation products, which is of particular relevance in the case of rapidly degrading ATX-a under natural conditions [93]. Such tests on environmental samples can provide useful information in cases of investigating sudden animal deaths after ATX-a has been degraded [94].

Other authors, to increase ATX-a selectivity and LODs, developed different methods using derivatization and fluorescence detection (FLD) [22,36,39,42,90,94,95]. The method developed by James and Sherlock [37] allowed for the detection of ATX-a in fresh water in Ireland and was used to relate this cyanotoxin to cases of neurotoxicosis in dogs [96]. A year later, the method was improved to allow for the simultaneous determination of ATX-a and HATX-a, as well as their degradation products (dihydroanatoxin-a, epoxyanatoxin-a, dihydrohomoanatoxin-a, and epoxyhomoanatoxin-a), with LODs below 10 ng/L and recoveries greater than 80% in water samples and cyanobacterial blooms [94]. In FLD, the excitation and emission wavelengths of ATX-a are frequently set at 470 and 530 nm, respectively [22,39,90]. However, Azevedo et al. [42] investigated the best detection conditions using different excitation and emission wavelengths, obtaining better results with a 480 nm excitation length and a 546 nm emission wavelength. Regarding derivatization, 4-fluoro-7-nitro-2,1,3-benzoxadiazole (NBD-F) is usually used, as it is a simple method that only takes a few minutes and exhibits selective reactivity with primary and secondary amines to form highly fluorescence derivatives [36,90,94,95]. Namera et al. [95] used the derivatization–solid-phase microextraction (SPME) technique coupled to HPLC-FLD to analyze ATX-a in aqueous simples. An improvement to this method was reported by Rellán and Gago-Martínez [39], who showed an LOD of 0.29 ng/mL in river water samples and changed the position of the derivatizing agent at the bottom of the reaction vial to extend the working life of the fiber. Thus, SPME is an effective alternative to conventional solid-phase extraction (SPE) for monitoring samples with a great number of analytes.

HPLC-FLD after derivatization with NBD-F has also been used for ATX-a analysis in more complex samples, such as algae-based dietary supplements [22,36], showing that the method can be robust to the matrix effect. In this case, the method allows for the determination of ATX-a and two of its degradation products (epoxyanatoxin-a and dihydroanatoxin-a) due to the use of o-phthaldialdehyde (OPA) and mercaptoethanol prior to the derivatization reagent [36].

In relation to multitoxin analysis, Vasas et al. [59] demonstrated the applicability of capillary zone electrophoresis (CZE) and micellar electrokinetic chromatography (MEKC) for the spectrophotometric detection (at 230 nm) of ATX-a, CYN, and MC-LR in water bloom samples and culture extracts. Low LODs were obtained for all three toxins (0.89–3.65 µg/mL), including ATX-a (2.63 µg/mL), proving to be a simple and rapid tool for testing the content of these cyanotoxins in environmental samples. However, for more complex samples, the authors recommended using both techniques for the confirmation of the results. Later, other authors applied HPLC-FLD for the analysis of ATX-a, β-methylamino-L-alanine (BMAA), and (2,4-diaminobutyric) DAB using a polymeric cation exchange solid-phase extraction (SPE) method in more complex matrices, as well as water samples (fish and aquatic plants) [63].

### 2.4. Mass Spectrometry Detection

The MS detection system is mainly based on the comparison of the specific analytical standards of the compound to be determined, being widely used for the determination of cyanotoxins [29]. Both the initial mass and the characteristic molecular ionic fragments are used to ensure the specificity of the assignment. However, in the case of ATX-a, it is important to note that this pattern is similar to that of phenylalanine, so it is necessary to know how this amino acid behaves when developing a method that allows for the detection of ATX-a with MS [97].

Ross et al. [98] were among the first research groups to use MS for ATX-a determination. They performed a comparison of different MS methods, obtaining the best results with the desorption chemical ionization/mass spectrometry tandem (DCI-MS/MS), with an LOD of pure ATX-a of 10 pg/µL. These authors used this method for the analysis of ATX-a in toxin-spiked urine samples, demonstrating that it could also be suitable for these types of matrices. However, it is important to note that, to date, it is not known whether ATX-a can be eliminated in urine, as the metabolism pathways for this toxin are still uncertain [1].

MS coupled to different techniques, such as LC or gas chromatography (GC), has been used for ATX-a determination in different matrices. Nevertheless, most of the methods developed employ LC-MS/MS when using water and cyanobacterial samples. To our knowledge, to date, only one study has focused on the analysis of fish samples using LC-MS/MS, achieving an LOD of 0.2 ng/g of ATX-a after carrying out an extraction by matrix solid-phase dispersion [37]. Moreover, the authors also evaluated the influence of the type of fish on the recovery rate of ATX-a, showing similar recovery values in all cases (around 75%). Dimitrakopoulus et al. [41] developed a LC-ESI-MS/MS method with the lowest reported LOD for the detection of ATX-a in freshwater samples using LC-MS/MS (LOD = 0.65 ng/L). To accomplish this, different types of SPE cartridges were also evaluated to select the most suitable one to carry out ATX-a preconcentration and extraction. Porous graphitized carbon cartridges showed the best recoveries compared to silica-based C18 or polymeric cartridges [41].

On the other hand, few studies employ GC-MS compared to LC-MS [22,34,38,99]. This may be due to the fact that the LODs achieved by this method are higher (µg/L ATX-a levels) than those obtained by LC-MS (ng/L ATX-a levels) and the fact that this method requires more analysis time due to the use of a derivatization process prior to the analysis. Rellán et al. [22] developed a method for the detection of ATX-a in dietary food supplements containing cyanobacteria, using GC-MS as a tool to confirm results obtained by HPLC-FLD. Although this technique has high sensitivity, as mentioned above, it can present errors in more complex matrices.

The use of other techniques coupled to MS has also been explored for ATX-a analysis. For instance, matrix-assisted laser desorption ionization time-of-flight (MALDI-TOF) has been performed for the characterization of ATX-a [40]; however, it does not indicate validation parameters for quantitative purposes. Also, laser diode thermal desorption–atmospheric pressure chemical ionization–tandem mass spectrometry (LDTD-APCI-MS/MS) was employed for the first time by Lemoine et al. [43] to identify ATX-a. This technique allows for the elimination of phenylalanine interferences without the need to use LC, thus reducing analysis time. Shortly thereafter, analytical data from this technique were improved using LDTD-APCI coupled with high-resolution mass spectrometry (HRMS) [46]. An LOD and a limit of quantification (LOQ) of up to 0.2 and 0.6 µg/L, respectively, were achieved using less than 15 s of analysis per sample.

More recently, other authors have used HRMS combined with direct analysis in real time (DART) for the analysis of ATX-a in cyanobacterial samples. This technique can be useful for the analysis of a large number of samples in a short time at the expense of the reproducibility of the method (around 30% RSD), with an LOD = 1 ng/mL [50]. Shortly afterwards, an improvement to this method was made using DART-HRMS/MS, which also allowed for the analysis of HATX-a and dihydroanatoxin-a, as well as the reduction of the matrix effect due to the use of an isotopically labeled ATX-a standard [53].

Regarding the methods for the determination of ATX-a in mixtures with other cyanotoxins, most of them have been validated using an MS detection system in different matrix samples. In addition to ATX-a, these methods have been validated to determine MCs (mainly MC-LR) and CYN, followed by other less common toxins such as STX, BMAA, DAB, NOD, okadaic acid (OA), and domoic acid (DA), among others. The first documented methods for the detection of ATX-a and MCs in water and fish muscle were developed by Hormozábal et al. [100,101], who obtained better validation parameters for the less complex matrix (water). Worse results were obtained by Pietsch et al. [102], who reported a very wide range of general toxin recoveries (3.2–96.0%) and a recovery of 50% for ATX-a after performing HPLC-ESI-MS/MS on water samples. They also determined STX and NOD. Subsequently, other authors improved these parameters by reducing the range of toxin recoveries (96–113%) and decreasing the LODs in phytoplankton samples with a higher number of toxins (STX, ATX-a, DA, NOD, six congeners of MCs, OA, and dinophysistoxin-1) [56]. These authors obtained the best overall toxin recovery parameters, with one of the highest ATX-a recoveries (103%) reported in this type of matrix, by LC-ESI-MS. Ortiz et al. [83] obtained similar parameters by LC-QtoF HRMS in cyanobacterial blooms, determining up to 13 congeners of MCs together with ATX-a.

In general, most of these methods have been developed for water samples, obtaining important differences in the validation parameters and showing wide ranges of toxin recoveries (recoveries of 35.5–107.5% were reported by Greer et al. [66]; recoveries of 65–138% were reported by Pekar et al. [67]; and recoveries of 44–113% were reported by Zervou et al. [71]. Among them, Pekar et al. [67] presented the best recoveries for ATX-a (range of 89–138%, compared to 35.5% [66] and 62.3% [71]) using UPLC-MS/MS and LC-MS/MS (see Table 2).

With respect to more complex matrices such as fish tissues, very few works have been published. As mentioned above, the first published report was from Hormozábal et al. [100], who obtained an ATX-a recovery of up to 73% using LC-MS. More recently, Haddad et al. [72] obtained a varied range of recoveries (45–103%) depending on each toxin, presenting a high percentage in the case of ATX-a (103%) using the same technique. Nevertheless, other authors have presented an overall high range of recoveries (83.2–109.8%) for all cyanotoxins, with 97.8% for ATX-a by LC-MS/MS [78]. However, when choosing a multitoxin method, it is very important to consider the recovery parameters of all toxins in general, in addition to those of ATX-a.

#### Method EPA 545

Because LC-MS is one of the most sensitive techniques, the Environmental Protection Agency (EPA) proposed this analytical method as the official method for the analysis of ATX-a in drinking water samples [103]. This guide indicates all the quality parameters that must be met by laboratories that wish to use this method for the detection of ATX-a and CYN. This method is a liquid chromatography, electrospray ionization, tandem mass spectrometry (LC/ESI-MS/MS) method. Thus, it requires the presence of analysts skilled in the operation of LC/ESI-MS/MS instrumentation and the interpretation of the associated data. Moreover, the minimum reporting levels for the lowest concentration in the laboratory were 0.018 μg/L for ATX-a and 0.063 μg/L for CYN.

The method describes the precision (95–111% for ATX-a) and accuracy (1.0–7.4% for ATX-a) values for ATX-a depending on the type of fortified water tested (water, groundwater, chlorinated surface water), as well as the influence of the contact packaging material (plastic and glass). Moreover, this method allows for the selection of LC columns and their conditions as long as they do not reduce analytical efficiency, and the analyst checks that all acceptance criteria for the quality control of the EPA-545 method are satisfied.

### 2.5. Gas Chromatography–Electron Capture Detection

GC coupled to electron capture detection (ECD) is also among the techniques used for ATX-a determination [104,105]. GC-ECD analysis of ATX-a requires the use of derivatization to increase the sensitivity of the analysis, resulting in a longer analysis time. To accomplish this, different derivatization processes have been employed, such as ones involving the use of trichloroacetic anhydride [104] or pentafluorobenzylbromide [105]. This technique was able to detect ATX-a levels up to pg levels in both water and cyanobacterial bloom samples [105].

## 3. General Discussion

The proliferation of toxin-producing cyanobacteria is increasing worldwide, with more and more variants of cyanotoxins being documented in the literature, as well as more alarming data regarding the concentrations of these toxins in samples. The development and validation of analytical methods is essential to alert the population to the presence of cyanotoxins and reduce potential exposure to cyanotoxins [106]. In recent years, cyanotoxins other than MCs, such as ATX-a, have attracted more attention [8]. Concentrations of up to 1430 µg/L and 8000 µg/g dw of ATX-a have been found in environmental samples [18,92,107]. Furthermore, a high bioaccumulation capacity has been demonstrated in fish [25,26].

On the other hand, different authors have demonstrated the presence of ATX-a or its derivatives in algae-based food supplements [108,109]. Therefore, because of the risk that the consumption of water and food contaminated with this toxin may pose, it is important to have adequate analytical methods to determine ATX-a. Furthermore, these methods should be in constant development to meet new analysis demands: the presence of cyanotoxins in different matrices, the coexistence of several toxins in one sample, different concentration ranges, etc.

**Figure 5:**
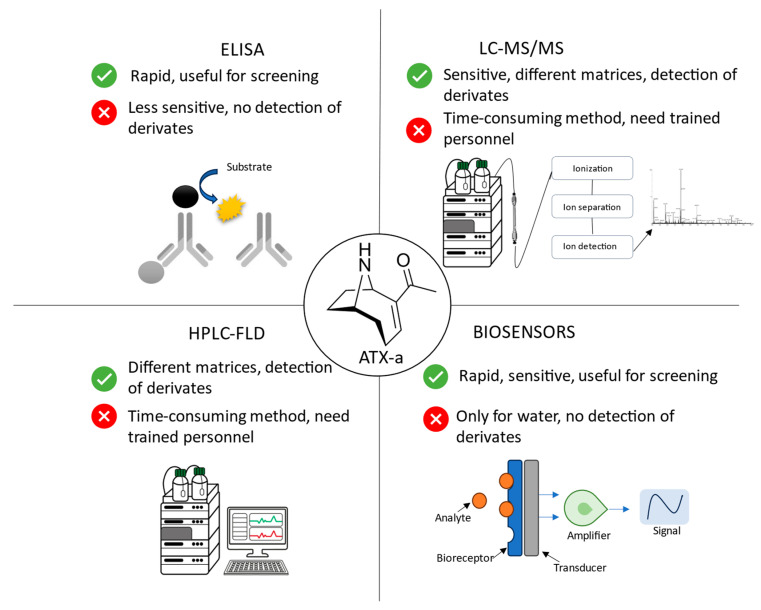
Advantages (green) and disadvantages (red) of the methods developed for ATX-a analysis. ELISA: enzyme-linked immunosorbent assay; HPLC-FLD: high-performance liquid chromatography coupled to fluorescence detection; LC-MS/MS: liquid chromatography–tandem mass spectrometry.

Several parameters must be taken into account when selecting an ATX-a analysis method. These include the availability of the analytical method, the type of matrix, the required analysis time, and the main validation parameters, such as the LOD, LOQ, % recovery, and precision. Figure 5 summarizes the main advantages and disadvantages of the analytical methods developed for ATX-a detection.

In general, although there are different methods for the detection of ATX-a alone, most of them use MS/MS detection systems [41,43,78,80]. These methods have shown the best validation parameters compared to other detection systems such as UV or FLD. With respect to the analysis of ATX-a alone in a water matrix, the method of analysis validated by Dimitrakopoulos et al. [41] using LC-ESI-MS/MS presents the best LOD levels (below 1 ng/L), a good range of toxin recoveries (73–97%), and adequate precision. However, methods using an MS/MS detection system require a prior cleaning, extraction, or preconcentration step, which increases the time of analysis, labor demands, and cost of the analytical process. A good alternative to them in this matrix (water) can be biosensors. The colorimetric biosensor validated by Nguyen and Jang [51] shows an LOD of 4.45 pM (equivalent to 0.7 ng/L) and a high recovery (89–112%).

More recently, rapid methods using DART-HRMS have been developed for cyanobloom samples; however, they have not shown good accuracy parameters (RSD% up to 34%) [50,53]. Recently, Cevallos-Cedeño et al. [54] developed an ELISA method for ATX-a. Although ELISA methods are fast and inexpensive methods for cyanotoxin analysis, they are less sensitive than other techniques. Nevertheless, they could be used as an alternative or screening method for an initial ATX-a analysis.

Regarding the methods used to analyze the presence of ATX-a in matrices other than water and cyanobacteria, we can find methods developed for fish [37,42] and algae-based supplements [22,108]. In fish tissue, the method of Bogialli et al. [37] presents a higher sensitivity, allowing for the detection of ATX-a at lower levels using LC-ESI-MS/MS, while the method developed by Azevedo et al. [42] presents a higher recovery rate and an RSD% < 1% when using HPLC-FLD. Considering the bioaccumulation of the above-mentioned toxin, it is essential to be able to develop new validated methods in different matrices (fish, plants, etc.) in order to carry out a correct risk assessment from a food safety point of view.

In recent years, multitoxin determination methods have attracted more interest than ATX-a alone analysis. This is because the presence of different cyanotoxins at the same time in field samples is becoming more and more frequent for different reasons. On the one hand, in nature, it is common to find different strains producing toxins that coexist in the same cyanobacterial bloom, while on the other hand, the same strain can lead to the production of different types of cyanotoxins [110]. Similarly to methods that focus on ATX-a alone, the analysis of ATX-a in combination with other cyanotoxins mainly uses MS/MS as the detection method.

Overall, multitoxin analytical methods show worse validation parameters (recovery rates, LOD, LOQ, and precision) than those focused on individual ATX-a analysis, despite most of them being more novel. This is largely due to the fact that the extraction process must consider the presence of both hydrophilic and hydrophobic toxins. However, we can find some multitoxin methods that show good recoveries of all the toxins analyzed (around 80–110%) [61,62,69,74]. These methods may be a good option for samples containing MCs [69], CYN [61], NOD [62], or even anabaenopeptins [74]. Regarding detection limits, the method of Filatova et al. [75] shows low detection levels for ATX-a, CYN, NOD, and MCs (order of ng/L), but it has the disadvantage of showing low recovery levels for some of the MC variants.

In relation to the type of matrix, water and cyanobacterial samples are also the matrices in which most analytical methods have been developed for cyanotoxin mixtures. However, fewer multitoxin determination methods have been developed in cyanoblooms compared to those for ATX-a. On the other hand, the number of multitoxin methods developed based on aquatic animals and vegetables is higher compared to the analytical methods for ATX-a alone. The method of Skafi et al. [78] can be applied for the analysis of ATX-a, HATX-a, CYN, 12 MC variants, anabaenopeptins A and B, and cyanopeptolin-A in fish tissue by LC-MS/MS, with recoveries between 83.2 and 109.8%. In addition, a validated method for spinach samples has recently been published that reports validation parameters [83]. This method uses capillary electrophoresis with tandem mass spectrometry (CE-MS/MS) to analyze MC-LR, MC-RR, CYN, ATX-a, NOD, DAB, BMAA, and AEG with good precision (%RSD < 12%) and LODs between 0.03 and 0.23 µg/kg. As evidenced by multitoxin methods with other matrices such as water [71,72,75], this method does not show high toxin recoveries (range 65.5–81.0%).

Nevertheless, although better toxin recovery data are obtained with ATX-a-only methods (more than 70%) [36,37,41,42], they may not be as practical for risk assessments. This is because ATX-a-only methods do not consider the possible presence of other cyanotoxins in samples, even those potentially more toxic and more abundant than ATX-a in natural samples, such as MC-LR and CYN.

Another important aspect of the analysis is to consider the possibility of degradation of ATX-a under environmental conditions. Methods that consider the determination of its degradation products allow for more realistic data on the status of this neurotoxin to be obtained. In this regard, there are different methods focused on the determination of ATX-a that can be applied [44,50,53,94]. However, there are few multitoxin methods that consider the detection of the products derived from ATX-a [60], meaning that, in cases in which this toxin is thought to be the main cause of poisoning, it may be advisable to use methods that only determine ATX-a and ATX-a-derived compounds.

Finally, in the determination of ATX-a, it is important to take into account its potential interference with phenylalanine, an amino acid present in foods rich in proteins. In this regard, several methods have been optimized to avoid erroneous results and false positives due to the presence of this amino acid in samples [41,43,46,48].

Taking all this into account, the selection of the most appropriate ATX-a analytical method in each case is multifactorial and must consider all the possibilities described above.

## 4. Conclusions

The need for rapid and reliable methods for the determination of ATX-a has led to the publication of a large number of articles focused on the analysis of both ATX-a alone and in mixtures with other cyanotoxins, with the multitoxin mode being highlighted in recent years. In general, the methods focused on ATX-a alone present a better sensitivity in determining this toxin. However, realistically, natural samples frequently contain several toxins.

In summary, bearing in mind the methods currently available for ATX-a analysis, to choose an appropriate method, it is necessary to consider the following: (1) the type of matrix; (2) whether it is likely to find more cyanotoxins as well as ATX-a and, if so, the types of these cyanotoxins; (3) the quality of the analytical parameters required; and (4) the cost and speed requirements. Most validated methods for the determination of ATX-a are based on the MS detection system and use simple matrices such as water samples or cyanobacterial blooms. Therefore, it is necessary to develop analytical methods for this toxin, both alone and in combination with other cyanotoxins, in more complex matrices (vegetables, crops, and fish).

## 5. Materials and Methods

### 5.1. The Information Sources and Search Strategy

Our search for information was conducted using research databases including Web of Science, Scopus, Science Database, and PubMed up to January 2024. The selected keywords for all search engines were as follows: anatoxin-a, cyanotoxins, detection methods, mass spectrometry. Furthermore, the bibliographies of the retrieved articles were reviewed to enhance the comprehensiveness of the search.

### 5.2. Inclusion and Exclusion Criteria

The following criteria were considered in the information selection process:

Inclusion criteria: (1) articles based on ATX-a determination methods providing optimization or new analytical validation parameters for ATX-a alone or in cyanotoxin mixtures; (2) articles published prior to January 2024; and (3) articles reporting comprehensive results published in internationally recognized journals.

Exclusion criteria: (1) articles on ATX-a determination that use methods already published by other authors and do not provide any new developments; (2) articles based on ATX-a(s) determination methods; (3) articles based on methods for the determination of cyanotoxin mixtures that do not consider the detection of ATX-a; (4) articles published in a language other than English; and (5) articles for which the abstract is only available.

Based on all the studies that complied with the inclusion criteria, different tables pertaining to the following were prepared: analytical methods focused on the determination of ATX-a published up to 2003 (Table S1) and from 2003 to date (Table 1), and analytical methods for the determination of cyanotoxin mixtures containing ATX-a published up to 2003 (Table S2) and from 2003 to date (Table 2). In addition, Figure 1, Figure 2 and Figure 3 were elaborated with the data contained in the articles of Table 1 and Table 2 and Supplementary Tables S1 and S2 considering all the ATX-a analysis works found.

Regarding the risk of bias due to the quality of the studies used for the analysis (Table S3), most of them showed a low (59.1%) or medium (37.8%) risk of bias, while only a minority (12.1%) demonstrated a high risk of bias, with these studies mainly being older in terms of date of publication and primarily focusing on the determination of AXT-a alone.

## Figures and Tables

**Table 1:** Analytical methods focused on the determination of ATX-a published from 2003 to date.

Type of Sample	Analytical Method	Linear Concentration Range	Validation Parameters	More Information	References
Cyanobacteria and water samples	LC-MS^n^	-	LOD < 0.6 µg/L Reproducibility (RSD%): ≤7% (LC-MS/MS and LC-MS^3^)	LC-MS^4^ did not produce reproductible quantitative data.	[32]
Cyanobacteria cultures	GC-MS ^1^H NMR	2–400 ng	LOD: 0.5 ng	Derivatization is not necessary. To improve the detection limits, SIM is necessary.	[33]
Water samples and cyanobacterial bloom	GC-MS	50–10,000 ng/L	LOD: 11.2 ng/mL LOQ: 200.1 ng/mL Intra-day (RSD%): 5.15% Inter-day (RSD%): 2.7%	Three forms of PANI films and a single form of PPY film were used, showing that the leucoemeraldine form of PANI displayed a better selectivity to ATX-a.	[34]
Cyanobacterial samples	QqTOF-MS QIT-MS	-	-	The investigated compounds were ATX-a, HATX-a, and their degradation products.	[35]
Food supplements (BGA and spirulina tablets, powders, and capsules)	LC-FLD	0.1–2.0 µg/g	LOD: 50 µg/kg (BGA) and 10 µg/kg (spirulina) Recovery: 74–108%	Derivatization reagent: NBD-F. Two transformation products of ATX-a were also analyzed: epoxyanatoxin-a (LOD: 10–55 µg/kg) and dihydroanatoxin-a (LOD: 10–65 µg/kg).	[36]
Water and fish muscle tissue	LC-MS/MS	-	LOD: 8 ng/L (water) and 0.2 ng/g (fish) LOQ: 13 ng/L (water) and 0.5 ng/g (fish) Recovery: 71–79%	Water samples were directly injected after filtration, whereas fish tissue required the matrix SPD technique. Provides sensitivity for analyzing ATX-a in fish at levels < 1 ng/g.	[37]
Water samples	GC-MS	2.5–200 ng/mL	LOD: 2.0 ng/mL LOQ: 2.5 ng/mL Repeatability (%RSD): 6.8–10.9%	Direct derivatization of the ATX-a by adding hexylchloroformate in a sample with pH = 9.	[38]
Water samples (deionized water and river water) and cyanobacterial samples	LC-FLD	6.25–1250 ng/mL	LOD: 0.18 ng/mL (deionized water) and 0.29 ng/mL (river water) Repeatability (RSD%): <15% Reproducibility (RSD%): <15%	Derivatization reagent: NBD-F SPME coupled to HPLC was used.	[39]
Cyanobacterial cultures	MALDI-TOF-MS	-	-	Derivatization reagent: N-methyl-N(tert-butyldimethylsilyl) trifluoroacetamide. Useful method for small sample volumes (1 µL) and has an analysis time of 1 min/sample.	[40]
Dietary food supplements containing cyanobacteria	LC-FLD	0.03–0.59 µg/mL	Recovery: 84% LOD: 3 ng/g LOQ: 10 ng/g Repeatability (%RSD): 6.3%	No significant effect on response of ATX-a in the matrix sample.	[22]
	GC-MS	5.87–23.50 µg/mL	LOD: 24 ng/g LOQ: 70 ng/g Repeatability (%RSD): 10.9%	13% of suppression of ATX-a response in sample matrix extract compared with solvent.	
Water samples	LC-MS/MS	0.5–2000 µg/L	Recovery: 73–97% LOD: 0.65 ng/L LOQ: 1.96 ng/L Intra-day (RSD%): 4.2–5.9% Inter-day (RSD%): 4.2–9.1%	Three different SPE cartridges were assessed—hypersep PGC SPE cartridges gave better recoveries. PHE-*d*_5_ was used as internal standard.	[41]
Water and lyophilized trout	LC-FLD	0.1–23.0 µg/mL	LOD: 0.17 µg/mL (water) and 80 ng/g (d.w. trout) LOQ: 0.58 µg/mL (water) and 170 ng/g (d.w. trout) Repeatability (RSD%): 0.4–0.9% Recovery: 84–94% Intermediate precision <2% Accuracy (RSD%): 6% (water) and <0.5% (trout)	Methanol is used as the mobile phase.	[42]
Cyanobacterial bloom	LDTD-APCI-MS/MS	3–250 µg/L	LOD: 1 µg/L LOQ: 3 µg/L Accuracy: 108% Inter-day (RSD%): 8%	Remove interference from PHE.	[43]
Cyanobacterial cultures	FP	0.1–200 µm	LOD: 33.3 nM LOQ: 100.0 nM	No effects of the solvent (methanol) in fluorescence intensity.	[44]
	LC-MS/MS	-	LOD: 1.5 ng/mL (5.33 nM) LOQ: 5.0 ng/mL (17.77 nM)	The sample was analyzed in positive MRM mode, searching for the transitions of the ATX-a and the most common ATX-a analogues: HATX-a, dihydroanatoxin-a, dihydrohomoanatoxin-a, epoxyanatoxin-a, and epoxyhomoanatoxin-a.	
Water samples	Aptasensor (DNA aptamers)	1–100 nM	LOD: 0.5 nM Recovery: 94.8–108.6%	The aptasensor exhibited high stability and selectivity of ATX-a against CYN and MC-LR.	[45]
Water samples	LDTD-APCI-HRMS	0.5–1000 µg/L	Recovery: 96–108% LOD: 0.2 µg/L LOQ: 0.6 µg/L Accuracy and precision values (RSD%) < 15%	Resolve the misidentification of ATX-a and PHE. Two scan modes were assessed, showing that targeted-MS/MS increased the selectivity of compound detection.	[46]
Cyanobacteria samples	LC-MS	0.005–1.25 µM	LOD: 0.02 mg/kg LOQ: 0.06 mg/kg Precision (RSD%): 1–12%	A novel mixed reverse-phase/weak anion exchange solid phase clean-up was employed. Also determines HATX-a (LOD: 0.04 mg/kg and LOQ: 0.12 mg/kg).	[47]
Water samples	IMS	20–150 µg/L	Recovery: 91–115% LOD: 0.02 µg/L LOQ: 0.08 µg/L Repeatability (RSD%): 3–9%	Interferences with PHE avoided using the d-MagIA extraction methodology.	[48]
Water samples	UPSS sensor	10^−15^–10^−10^ M	LOD: 10^−14^ M	Selectivity of ATX-a against CYN and brevetoxin-2.	[49]
Cyanobacterial samples	DART-HRMS	0.283–206 ng/mL	LOD: 1 ng/mL Intra-day (RSD%): 10–40% Inter-day (RSD%): 33%	<2 min of analysis per sample for triplicate analysis. Also determines HATX-a and dihydroanatoxin-a.	[50]
Water samples	Colorimetric biosensor (DNA aptamer)	10 pM–200 nM	LOD: 4.45 pM Repeatability (RSD%): 3.6% Recoveries: 89.72–112.43% (RSD: 4.12–10.91%)	Gold nanoparticles are used as probes. Excellent specificity toward ATX-a.	[51]
Water samples	ECL-RET aptasensor	0.001–1 mg/mL	LOD: 0.00034 mg/mL	This sensor showed high assay performance for ATX-a determination.	[52]
Cyanobacterial samples	DART-HRMS/MS	0.14–86 ng/mL	LOD: 4.8 ng/g cyanobacteria Recovery: 82–84% Precision (RSD%): 12–34%	Also determines HATX-a and dihydroanatoxin-a.	[53]
Water samples	ELISA (direct and indirect)	0.5–500 ng/mL	Direct and indirect ELISA: LOD: 0.1 ng/mL Recovery: 82–117.4% CV < 20% Intra-day (RSD%) < 10% Inter-day (RSD%) < 20%	Detected (+)-ATX-a. Robust to pH variations. It is advisable to minimize the concentration of organic solvents, and PBS proved to be the most suitable buffer for the ELISA.	[54]
	LFICA	-	LOD: 2 ng/mL	No influence of pH.	
Cyanobacterial samples	LC-HRMS/MS	-	-	Allows for the detection of new ATX-a derivatives.	[55]

APCI: atmospheric pressure chemical ionization; ATX-a: anatoxin-a; BGA: blue–green algae; CV: coefficients of variation; DART: direct analysis in real time; CYN: cylindrospermopsin; DNA: deoxyribonucleic acid; d-MagIA: dispersive magnetic immunoaffinity; d.w.: dry weight; ECL-RET: electrochemiluminescence resonance energy transfer; ELISA: enzyme-linked immunosorbent assay; FLD: fluorescence detection; FP: fluorescent polarization; GC: gas chromatography; HATX-a: homoanatoxin-a; ^1^H NMR: proton nuclear magnetic resonance; HPLC: high-performance liquid chromatography; HRMS: high-resolution mass spectrometry; IMS: ion mobility spectrometry; LC: liquid chromatography; LDTD: laser diode thermal desorption; LFICA: lateral flow immunochromatography assay; LOD: limit of detection; LOQ: limit of quantification; MALDI-TOF-MS: matrix-assisted laser desorption ionization time of flight mass spectrometry; MC-LR: microcystin-LR; MRM: Multiple Reaction Monitoring; MS/MS: tandem mass spectrometry; MS^n^: multiple tandem mass spectrometry; NBD-F: 7-Fluoro-4-nitro-2,1,3-benzoxadiazole; PANI: polyaniline; PBS: phosphate-buffered saline; PGC: porous graphitic carbon; PHE: phenylalanine; PPY: polypyrrole; QIT: quadrupole ion-trap; QqTOF: hybrid quadrupole time-of-flight; RSD: relative standard deviation; SIM: selected ion monitoring; SPD: solid-phase dispersion; SPE: solid-phase extraction; SPME: solid-phase microextraction; UPSS: ultrasensitive polymeric sensing system.

**Table 2:** Analytical methods for the determination of cyanotoxin mixtures containing ATX-a published from 2003 to date.

Type of Sample	Cyanotoxins	Analytical Method	Linear Concentration Range	Global Validation Parameters of Multitoxin Methods	More Information and Specific ATX-a Data	References
Phytoplankton and aqueous phytoplankton	STX, ATX-a, DA, NOD, MCs (-RR, -YR, -LR, LA, -LW, and -LF), OA, and DTX-1	LC-MS	1 to 50 ng on column	LOD: 0.5 to 1.0 ng RSD%: 0.6–7.1% Recoveries: 96–113%	Direct analysis after the simple preparation of the sample. The applied chromatographic conditions allow for the isolation and identification of substances suspected to be “new” microcystins. For ATX-a: LOD: 0.5 ng, RSD%: 3.2%, and recovery: 103%.	[56]
Cyanobacterial cultures	STX and its various analogues, ATX-a, CYN, doCYN, MCs (-LR and -RR)	LC-MS	-	LOD: 1.4–3.2 pmol injected on column in SIM mode	MS detection was carried out in the SIM and SRM modes for the positive ions, with SRM being the best performing. In samples, a simple extraction method was used, and no clean-up was performed on the crude extracts in order to demonstrate rapid analysis. For ATX-a: LOD: 1.4 pmol injected on column in SIM mode.	[57]
Water samples	ATX-a, MCs (-LR, -RR, and -YR), and NOD	LC-MS	5–50 µg/mL	LOD: less than 1 µg/L RSD%: 5–19% Recoveries: 68–98%	Toxins are partitioned from water samples with extraction disks. It allows for sample analysis, including processing in 1 h. Does not provide multipoint linearity data due to the lack of sufficient quantities of analyte. For ATX-a: LOD: less than 1 µg/L, and recovery: 68%.	[58]
Water bloom samples and crude cyanobacterial extracts	ATX-a, MC-LR, and CYN	CZE-UV	-	LOD: 0.73–3.77 µg/mL RSD% peak area: 1.48–2.93% RSD% migration time: 0.27–0.71%	For ATX-a: LOD: 1.12 µg/mL; RSD% peak area: 2.93%; and RSD% migration time: 0.27%.	[59]
		MEKC-UV	-	LOD: 0.89–3.65 µg/mL RSD% peak area: 1.87–2.98% RSD% migration time: 0.58–0.74%	It is recommended to use both CZE and MEKC for the analysis of the same sample in order to confirm the results. For ATX-a: LOD: 2.63 µg/mL; RSD% peak area: 2.98%; and RSD% migration time: 0.66%.	
Cyanobacteria-containing samples	PSP toxins, ATXs, CYNs, MCs (-RR, -LR, -YR, -LA, -LW, and -LF), and NODs	LC-MS/MS	-	LOD: 6–700 pg LOQ: 10–1150 pg	It is necessary to clean the sample to avoid false positives. This method allows one to detect potential unknown variants of cyanobacterial toxins. For ATX-a: LOD: 700 pg and LOQ: 1150 pg	[60]
Water samples	ATX-a, MCs (-RR, -LR, and -LF), and CYN	LC-MS/MS	0.5–100 ppb (µg/L)	LOD: 0.10–0.21 ppb (µg/L) Recoveries: 88–110%	This method can be used to either screen or quantify the main cyanotoxins in a few minutes. For ATX-a: LOD: 0.13 µg/L and recoveries: 88–102%.	[61]
Water samples	MCs (-LR, -RR, -YR, -LW, -LF, and -LA), NOD, ATX-a, and CYN	LC-MS	8–1000 ng/L (MCs and NOD) 40–2000 ng/L (ATX-a) 40–2000 ng/L (CYN)	LOD: 2–100 ng/L (pure water) Recoveries: 83–104%	Dual-cartridge SPE extraction is necessary. Toxin recoveries were lower in reservoir water than in pure water (83–90% vs. 94–104%). Differences of less than 10% were obtained in the values of MC-LR and CYN concentrations measured by this method (SPE-LC-MS) compared to the ELISA test. For ATX-a (pure water): LOD: 46 ng/L and recovery: 96%.	[62]
Water, fish, and plant samples	BMAA, DAB, and ATX-a	LC-FLD	0.01–0.70 mg/L (BMAA and ATX-a) 0.01–1 mg/L (DAB)	LOD: 5–7 µg/L	Derivatization reagent: 6-aminoquinolyl-N-hydroxysuccinimidyl carbamate. An extraction step with SPE cartridges is required. The sensitivity provided by LC-MS/MS was improved over HPLC-FLD. For ATX-a: LOD: 6 µg/L in HPLC-FLD and 3.2 µg/L in LC-MS/MS.	[63]
		LC-MS/MS	0.01–0.70 mg/L (BMAA and ATX-a) 0.01–1 mg/L (DAB)	LOD: 0.8–3.2 µg/L		
Water samples	CYN, MCs (-LR, -RR, and -YR), and ATX-a	LC-MS/MS	-	LOD: 1.9–3.9 ng/mL LOQ: 5–10 ng/mL	The method separates the amino acid PHE, so there is no interference with ATX-a. The most common cyanotoxins can be simultaneously identified within 12.5 min, including several ATX-a analogues. For ATX-a: LOD: 1.9 ng/mL and LOQ: 5 ng/mL.	[64]
Algal bloom water samples	MCs (-RR, -YR, -LR, -LY, -LW, and -LF), ATX-a, and CYN	LC-MS/MS	0.1–10 µg/L	LOD: 0.01–0.02 µg/L LOQ: 0.03–0.06 µg/L Intra-day (RSD%): 3–9% Inter-day (RSD%): 7–13% Bias (%): 7–12% Recoveries: 72–102%	SPE extraction is required. The analysis time was 7 min per sample. The method permitted the chromatographic separation of ATX-a and PHE. For ATX-a: LOD: 0.01 µg/L, LOQ: 0.03, bias: 8–9%, intra-day (RSD%): 4–6%, inter-day (RSD%): 9–13%, and recovery: 72%.	[65]
Aquatic samples	MCs (-LR, -YR, -RR, -LA, -LY, and -LF), NOD, CYN, ATX-a, and DA	LC-MS/MS	0.01–2 ng/mL	LOD: 0.3 and 5.6 ng/L LOQ: 0.8 and 18.5 ng/L Intra-day (RSD%): 1.2–9.6% Inter-day (RSD%): 1.3–12.0% Recoveries: 35.5–107.5%	SPE extraction is required. Very low recoveries are obtained for ATX-a and DA (35.5 and 65.5%, respectively). The rest of the toxins show good recoveries (93.8–107.5%). For ATX-a: LOD: 5.6 ng/L, LOQ: 18.5, ng/L: 1.3–12.0%, and recoveries: 35.5%.	[66]
Water samples	ATX-a, HATX-a, CYN, doCYN, NOD, and MCs (-LR, -RR, -YR, HtyR, -LA, -LF, -LW, -LY, -WR, -[Dha7]-LR, -[d-Asp3]-LR, -[d-Asp3]-RR, -HilR, -(*N*-methyl-l)-R, -[d-Asp3, (E)-Dhb7]-HphR, -[d-Asp3, (E)-Dhb7]-HtyR, -[d-Asp3, (E)-Dhb7]-RR, -[Dha7]-LR)	LC-MS/MS	0.1–10 µg/L	LOQ: 0.1–0.5 µg/L RSD%: 0.9–5.4% Recoveries: 60–111% for drinking water Recoveries: 65–138% for raw water	The time of analysis, including the lysis of cell-bound toxins, is less than three hours. For ATX-a: LOQ: 0.5 µg/L and recoveries: 89–138% (raw water).	[67]
Drinking water samples	MCs (-RR, -YR, -LR, -WR, -LA, -LY, -LW, and -LF), NOD, ATX-a, and CYN	LC-MS	0.1–10 µg/L (ATX-a, MC-RR, -WR, -LA, -LF, and NOD) 0.2–20 µg/L (MC-YR, and -LW) 0.3–30 µg/L (CYN, MC-LR, and -LY)	LOD < 100 ng/L (range of 30–90 ng/L) LOQ: 0.1–0.3 µg/L RSD < 20% (intra-day (RSD%): 5.1–16.6% and inter-day (RSD%): 5.8–13.7 for 1 µg/L; intra-day (RSD%): 2.5–16.0% and inter-day (RSD%) 4.6–12.3 for 200 ng/L) Accuracy: intra-day 93–107% and inter-day: 84–99% for 1 µg/L; accuracy intra-day: 89–106% and inter-day 91–105 for 200 pg/mL	Adequate separation of toxins in 12 min. The matrix effects between calibrators and samples (≤30%) were negligible. For ATX-a: LOD: 30 ng/L and RSD < 20% (intra-day (RSD%): 8.6% and inter-day (RSD%) 8.1% for 1 µg/L; intra-day (RSD%): 5.1% and inter-day (RSD%) 6.3 for 200 ng/L; accuracy: intra-day 101% and inter-day: 99% for 1 µg/L; and accuracy intra-day: 106% and inter-day 105% for 200 pg/mL	[68]
Cyanobacterial blooms samples	MCs (-LR, -YR, -RR, -HtyR, -HilR, -WR, -LW, -LA, -LF, -LY, -Dha7, -LR, and -Dha7-RR) and ATX-a	LC-HRMS	0.05–4.80 µg/L	LOD: 0.004 and 0.01 μg/L Intra-day (RSD%): 1.71–4.68% Inter-day (RSD%): 1.95–3.63% Recoveries: 93.5–105.4%	Automated preconcentration of the sample (on-line SPE) was used. This method presents quantitative results in a period less than 3 h. Uncertainty of method: 4 and 14%. For ATX-a: LOD: 0.0044 µg/L, intra-day (RSD%): 3.66%, inter-day (RSD%): 3.42%, recovery: 98.9%.	[69]
Algal dietary supplements (Spirulina and *Aphanizomenon flos-aquae*)	MCs (-RR, -YR, -LR, -LA, -LY, -LW, and -LF), ATX-a, dihydroanatoxin-a, epoxyanatoxin-a, CYN, STX, and BMAA	LDTD-APCI-HRMS and LC-HRMS	0.03–20 µg/g	LODs: 0.01–0.1 µg/gLOQs: 0.03–0.1 µg/gIntra-day (RSD%): 1–9% and 5–12% for high and low concentrations Inter-day (RSD%): 7–13% and 7–15% for high and low concentrations Recoveries: 79–97%	The ATX-a recoveries obtained using LDTD-APCI-HRMS analysis were better (92–95%) than applying UHPLC-HESI-HRMS analysis (89–90%). However, a better linearity range and better LOD and LOQ data were obtained with UHPLC-HESI-HRMS compared to LDTD-APCI-HRMS. For ATX-a: LOD: 0.04 µg/g, LOQ: 0.1 µg/g, intra-day (RSD%): 5%, inter-day (RSD%): 8–9%, and recovery: 89–90%.	[70]
Water samples	CYN, ATX-a, NOD, MCs ([D-Asp3]-RR, -RR, -YR, -HtyR, [D-Asp3]-LR, -LR, -HilR, -WR, -LA, -LY, -LW, and -LF), OA, and DA	LC-MS/MS	1–250 µg/L	LODs: 1–10 ng/L RSD%: 5.5–45.8% Recoveries: 44–113%	In general, the mean recoveries and precision parameters are in agreement with guidelines for all common toxins. The worst analytical validation data (recovery and precision) were found for MC-WR, MC-LF, and MC-LW. Nevertheless, the decreased efficiency of the method, observed only for these analytes, is compensated for by the advantage of achieving the simultaneous determination of numerous toxins. For ATX-a: LODs: 1 ng/L, RSD%: 25.38%, and recovery: 62.3%.	[71]
Water samples and fish tissue	MCs (-LA, -LR, -LY, -RR, and -YR), NOD, ATX-a, CYN, and STX	LC-MS	0.1–100 ng/mL 0.1–20 ng/mL (ATX-a)	LOD: 0.004–0.080 ng/mL LOQ: 0.01–0.28 ng/mL RSD% water: 0.6–7.2% RSD% fish tissue: 1.2–8.2% Recoveries water: 53–98% Recoveries fish tissue: 45–103%	SPE extraction was used for water, and liquid–liquid extraction was used for fish tissue. A zwitterionic HILIC was evaluated to separate toxins. For ATX-a: LOD: 0.004 ng/mL, LOQ: 0.01 ng/mL, RSD% water: 1.9%, RSD% fish tissue: 3.4%, recovery water: 97%, and recovery fish tissue: 103%.	[72]
Water samples	ATX-a, CYN, NOD, and MC-LR	Differential fluorescent sensor array (DNA aptamer)	8–100,000 nM	LOD of fluorimeter: 0.54–1.8 nM LOD of smartphone: 1.2–2.8 nM LOQ: 8–23 nM Recoveries: 97–104.6% RSD%: 0.9–3%	The smartphone-based sensor platform showed remarkable chemical specificity against potential interfering agents in water. This assay chip runs to completion after the addition of a drop of environmental water, making it suitable for field applications. Robust to various interferences (minerals, organics, and biomacromolecules). For ATX-a: LOD of fluorimeter: 0.54 nM and LOD of smartphone: 1.2 nM.	[73]
Water samples	CYN, ATX-a, HATX-a, AP-A and AP-B, and MCs (-RR, [Asp3]-RR, -YR, -HtyR, -LR, [Asp3]-LR, -HilR, -WR, -LA, -LY, -LW, and -LF)	LC-HRMS	-	LOD: 8 and 53 ng/L LOQ: 36 and 176 ng/L Intra-day (RSD%): 1.2–17% Inter-day (RSD%): 3.1–19% RSD% < 20% Recoveries: 81–113%	On-line SPE extraction was used. Extraction and separation of toxins were achieved in 8 min. Low relative matrix effects (<29%). For ATX-a: LOD: 15 ng/L and LOQ: 49 ng/L.	[74]
Water reservoirs	CYN, ATX-a, NOD, and MCs (-LR, -RR, -YR, -LA, -LY, -LW, -LF).	LC-HRMS	0.025–50 µg/L	LOD: 4 and 150 pg/L LOQ: 12 and 450 pg/L Intra-day (RSD%): 1.5–8.8% Inter-day (RSD%): 2–23.2% Recoveries: 9.2–84.3% at 2 ng/L Recoveries: 32.3–70.3% at 10 ng/L Recoveries: 48.7–87.8% at 20 ng/L	Two-step SPE extraction was used. The best recoveries of 70.2–87.8% were obtained for ATX-a, and the worst data recoveries (only 9.2–48.7%) were obtained with MC-LW. For ATX-a: LOD: 20 pg/L, LOQ: 60 pg/L, intra-day (RSD%): 2.1%, inter-day (RSD%): 22.6%, and recoveries: 70.2–87.8%.	[75]
Cyanobacterial bloom freshwater	CYN, ATX-a, and HATX-a	LC-MS/MS	0.03–100 ng/mL	LOD by DI: 15–70 ng/L LOD by SPE: 0.6–1.3 ng/L LOQ by DI: 50–240 ng/L LOQ by SPE: 2–4 ng/L RSD%: 1.4–3.3%	ATX-a and HATX-a in freshwater samples could be performed by both DI and SPE coupled with UPLC-ESI-MS/MS; the best values were obtained by SPE. Acetaminophen-d4 (an isotopically labeled acetaminophen) is a suitable internal standard for correcting the matrix effects on the signal intensity of ATX-a and HATX-a. For ATX-a: LOD by DI: 70 ng/L, LOD by SPE: 1.3 ng/L, LOQ by DI: 240 ng/L, LOQ by SPE: 4 ng/L, and RSD%: 2.2%.	[76]
Water samples	ATX-a, HATX-a, CYN, and MCs (-LF, -LR, -RR, -YR, [D-Asp3]-LR, [D-Asp3]-RR)	LC-HRMS	0.02–100 µg/L	LOD extracellular: 10–129 ng/L LOD intracellular: 3–45 ng/L LOQ extracellular: 25–129 ng/L LOQ intracellular: 8–45 ng/L Intra-day (RSD%): 2–7% Inter-day (RSD%): 2–7% Recoveries:84–119%	Matrix effects for the intracellular fraction were similar and acceptable for all analytes. For ATX-a: LOD extracellular: 10 ng/L, LOD intracellular: 3 ng/L, LOQ extracellular: 25 ng/L, LOQ intracellular: 8 ng/L, intra-day (RSD%): 2–4%, inter-day (RSD%): 4%, and recoveries: 84–100%	[77]
Fish muscle	ATX-a, HATX-a, CYN, 12 MCs, AP-A and AP-B, and cyanopeptolin-A	LC-MS/MS	0.05–250 µg/kg	LOD: 0.3–10 µg/kg LOQ: 0.3–33 µg/kg Intra-day (RSD%): 1.8–11.4% Inter-day (RSD%): 7.2–25.5% Recoveries: 83.2–109.8%	For the ATX-a, the parameters were as follows: LOD: 10 µg/kg, LOQ: 33 µg/kg, intra-day (RSD%): 1.8%, inter-day (RSD%): 19%, and recovery: 97.8%.	[78]
Water reservoirs	MCs (-LR and -RR), NOD, CYN, ATX-a, BMAA, DAB, and AEG	LC-MS/MS	0.004–2.00 µg/L	LOD: 0.0012–0.03 µg/L LOQ: 0.004–0.1 µg/L Intra-day (RSD%): 3.3–14.1% Inter-day (RSD%): 5.1–14% Recoveries: 70.6–101.0%	Matrix effects: 11.2–253%. Different types of stationary phases were tested. The results showed that the Oasis MCX cartridge provided satisfactory recoveries for all cyanotoxins (above 55%), with the exception of ATX-a. Moreover, different membrane filters (CA, GF, PTFE, Nylon, and PVDF) were studied, with the hydrophilic PTFE filter membrane being the most reliable filter material. Separation and detection of toxins were achieved in less than 12 min. For ATX-a: LOD: 0.03 µg/L, LOQ: 0.1 µg/L, intra-day (RSD%): 3.6–4.5%, inter-day (RSD%): 6.4–14%, recoveries: 82.3–92.8%, and matrix effect: 11.4–16.8%.	[79]
Algal dietary supplements (Spirulina)	MCs (-LR and -RR), NOD, ATX-a, BMAA, DAB, and AEG	LC-MS/MS	60–2500 µg/kg	LOD: 15–90 µg/kg LOQ: 50–300 µg/kg Intra-day (RSD%): 3.7–19.5% Inter-day (RSD%): 5.6–25.1% Recoveries: 64.2–102.9%	Matrix effects: 9.1–467.8%. For ATX-a, matrix effects: 26–33%. For ATX-a: LOD: 45 µg/kg, LOQ: 150 µg/kg, intra-day (RSD%): 7.1–9.1%, inter-day (RSD%): 8.2–8.6%, and recoveries: 78.7–87.8%.	[80]
Shellfish	STXs, ATX-a, CYN, NOD doCYN, DTXs, DA, MCs, BMAA, DAB, AEG, azaspiracids, yessotoxins, spirolides, pectenotoxins, pinnatoxins, ovatoxins, gymnodimines, and brevetoxins	LC-MS/MS	-	LOD: 1.2–150 µg/kg LOQ: 3.5–450 µg/kg Recoveries: 48–107.5%	For ATX-a: LOD: 8 µg/kg, LOQ: 17.5 µg/kg, and recovery: 94%.	[23]
Bivalve mollusks and phytoplankton	ATX-a, HATX-a, CYN, MCs (-RR[D-Asp3], -RR[D-Asp3, (E)-Dhb7], -LA, -LR-[Dha7], -LR-[Asp3], -LF, -LR, -LY, -HilR, -LW, -YR, -HtyR, and -WR), and NOD	LC-MS/MS	3.12–200 µg/kg	LOD: 2.1–4.04 µg/kg LOQ: 6.29–12.11 µg/kg Within-batch repeatability (RSD%): 1.3–7.4%; between-batch repeatability (RSD%): 5.6–18% Recoveries: 18.6–88.8%	ATX-a, HATX-a, MC-LF, MC-LW, and especially CYN presented the worst recovery data. For ATX-a: LOD: 2.51 µg/kg, LOQ: 7.52 µg/kg, and recoveries: 57.9–60.6%.	[81]
Cyanobacterial samples	ATX-a, HATX-a, CYN, doCYN, NOD, GNT, MCs (-RR, [C-Asp3]RR, -LA, -LR, -LY, -LW, and -YR), and STXs	LC-MS/MS	-	-	First method to detect GNT simultaneously with other cyanotoxins.	[82]
Water samples	MCs (-LR and -RR), NOD, CYN, ATX-a, DAB, BMAA, and AEG	CE-MS/MS	0.03–2.5 µg/L (NOD and CYN) 0.02–0.15 µg/mL (rest of cyanotoxins)	LOD: 0.005–0.102 µg/L LOQ: 0.016–0.340 µg/L Recoveries: 53.5–105% Intra-day (RSD%): <9.8% Inter-day (RSD%): <13.7%	No significant matrix effect. The lower recoveries were obtained for DAB. For ATX-a: LOD: 0.005 µg/L and LOQ: 0.016 µg/L.	[83]
Spinach samples			0.6–2.4 µg/kg (AEG, DAB, and MC-LR) 0.45–1.80 µg/kg (ATX-a, MC-RR, and BMAA) 1.28–8 µg/kg (NOD and CYN)	LOD: 0.03–0.23 µg/kg LOQ: 0.10–0.78 µg/kg Recoveries: 65.5–81.0% Precision (RSD%): 1.1–11.9%	For ATX-a: LOD: 0.03 µg/Kg and LOQ: 0.10 µg/kg.	

AEG: N-(2-aminoethyl)glycine); APs: anabaenopeptins; APCI: atmospheric pressure chemical ionization; ATX-a: anatoxin-a; BMAA: β-methylamino-L-alanine; CA: cellulose acetate; CYN: cylindrospermopsin; CZE: capillary electrophoresis; DA: domoic acid; DAB: 2,4-diaminobutyric acid; DI: direct injection; DNA: deoxyribonucleic acid; doCYN: deoxycylindrospermopsin; DTX-1: dinophysistoxin-1; ELISA: enzyme-linked immunosorbent assay; ESI: electrospray ionization; FLD: fluorescence detection; GNT: guanitoxin; GF: glass microfiber; HATX-a: homoanatoxin-a; HESI: heated electrospray ionization source; HPLC: high-performance liquid chromatography; HRMS: high-resolution mass spectrometry; LC: liquid chromatography; LDTD: laser diode thermal desorption; LOD: limit of detection; LOQ: limit of quantification; MEKC: micellar electrokinetic chromatography; MC: microcystin; MS: mass spectrometry; MS/MS: tandem mass spectrometry; NOD: nodularin; OA: okadaic acid; PHE: phenylalanine; PTFE: polytetrafluoroethylene; PSP: paralytic shellfish poisoning; PVDF: polyvinylidene fluoride; RSD: relative standard deviations (precision, repeatability, and reproducibility); SIM: selected ion monitoring; SPE: solid-phase extraction; SRM: selected reaction monitoring; STX: saxitoxin; UHPLC: ultra-high-performance liquid chromatography; UPLC: ultra-performance liquid chromatography.

## Data Availability

The data presented in this study are available in this article and Supplementary Materials.

## Supplementary Materials

The following supporting information can be downloaded at: https://www.mdpi.com/article/10.3390/toxins16040198/s1. Table S1: Analytical methods focused on the determination of ATX-a up to 2003 [111]; Table S2: Analytical methods focused on the determination of cyanotoxin mixtures containing ATX-a up to 2003. Table S3: Risk of bias for the methodological quality of studies reporting different analytical methods for ATX-a determination—0: not reported; 1: not appropriately or clearly evaluated; 2: appropriately evaluated. M: medium (4–6); L: low (7–8); H: high (0–3).
