# Supplementary material for: Analytical Methods for Anatoxin-a Determination: A Review
Source: Toxins (Basel). 2024 Apr 19;16(4):198. doi: 10.3390/toxins16040198 (PMC11053625; doi:10.3390/toxins16040198)
Supplement: Supplementary file 1 [file toxins-16-00198-s001.zip › toxins-2886975-supplementary.pdf]

Table S1. Analytical methods focused on the determination of ATX-a up to 2003.

Type of sample	Analytical method	Linear concentration range	Validation parameters	More information	References
Cyanobacterial cultures	HPLC-UV	-	-	-	[87]
Cyanobacterial cultures	GC-ECD	8.7-87.0 ng/extract	-	Internal standard: (±)-sec-butylnipecotate<br>Derivatization reagent: trichloroacetic anhydride.	[104]
Cyanobacterial cultures	TLC-UV<br>HPLC-UV	HPLC: 20-100 ng	-	-	[30]
Cyanobacterial bloom and water samples	GC-MS	1-20 mg Anabaena/20 mg sample	LOD: 5 μg/g	-	[99]
Cyanobacterial cultures and urine samples	DCI-MS/MS	10-10000 pg	LOD: 10 pg/μL	Different MS methods were evaluated, with DCI-MS being the method with the best results. Isobutane or ammonia DCI-MS can be used to detect ATX-a. Moreover, it is a simple method that does not require an extraction step.	[98]
Cyanobacterial cultures	TLC	10-100 μg/g	LOD: 10 μg/g	Facile high-capacity screening.<br>No clear interfering spots in the vicinity of ATX-a.	[31]
Cyanobacterial bloom	HPLC-UV	0.084-3.820 μg/mL	LOD: 0.8 ng<br>Precision (RSD%): 1.1-1.4%<br>Inter-day (RSD%): 2.4%<br>Recovery: 90.9-100.0%	Used an isocratic ion-pair reversed-phase HPLC method. Determined also HATX-a.	[88]
Cyanobacterial cultures	HPLC-UV<br>GC-MS<br>CE	HPLC-UV: 0.07-5.40 μg/mL<br>CE: 1.60-8.00 μg/mL	-	Determined also HATX-a and propylanatoxin.	[89]

Cyanobacterial cultures	GC-ECD	-	LOD: 2.5 pg	Derivatization reagent: pentafluorobenzylbromide.	[105]
Water samples	HPLC-FLD	1-20 μg/L	LOD: 0.1 μg/L<br>Recovery: 83-97%	Derivatization reagent: NBD-F.	[90]
Water samples (raw and treated water)	HPLC-UV	1-5 μg/L	Response factor (peak area/concentration)<br>RSD: 1.30%<br>LOD: 0.025 μg/L	Used extraction with styrene-divinylbenzene copolymer sorbent.	[91]
Water samples and cyanobacterial bloom	HPLC-FLD	-	Recovery: 83.2-84.9%<br>RSD%: 1.7-3.9%<br>LOD: < 10 ng/L	Derivatization reagent: NBD-F<br>The investigated compounds were ATX-a, HATX-a and their degradation products: dihydroanatoxin-a, epoxyanatoxin-a, dihydrohomoanatoxin-a and epoxyhomoanatoxin-a.	[94]
Water samples	LC-ESI-MS	5-5000 ng/L	Recovery: 75.7%<br>RSD%: 7.2%<br>LOD: 2.1 ng/L<br>LOQ: 15.2 ng/L	Automated on-line derivatization procedure with fluorenyl methylchloroformate.<br>ATX-a extracted using SPE.	[111]
Food supplement (Spirulina tablet and capsule samples)	SRM micro-LC-MS/MS	0.2-2.5 μg/mL	-	The investigated compounds were ATX-a, HATX-a and their degradation products: dihydroanatoxin-a, epoxyanatoxin-a, dihydrohomoanatoxin-a and epoxyhomoanatoxin-a.	[108]
Water samples	HPLC-FLD	50-1500 ng/mL	LOD: 20 ng/mL<br>Intra-day (RSD%): 7.6%	Derivatization reagent: NBD-F.<br>SPME coupled to HPLC was used.<br>Good repeatability was obtained by two different derivatizing addition method: microsyringe and spray procedure.	[95]

ATX-a: anatoxin-a; CE: capillary electrophoresis; DCI: desorption chemical ionization; ECD: Electron capture detector; ESI: electrospray ionization; FLD: fluo-rescence detection; GC: gas chromatography; HATX-a: homoanatoxin-a; HPCL: high-performance liquid chromatography; LOD: limit of detection; LOQ: limit of quantification; MS/MS: tandem mass spectrometry; NBD-F: 7-

Fluoro-4-nitro-2,1,3-benzoxadiazole; RSD: relative standard deviation; SPE: solid-phase extraction; SPME: solid-phase microex-traction; SRM: selected reaction monitoring; TLC: thin layer chromatography; UV: ultraviolet

Table S2. Analytical methods focused on the determination of cyanotoxins mixtures containing ATX-a up to 2003.

Type of sample	Cyanotoxins	Analytical method	Linear concentration range	Global validation parameters of multitoxins methods	More information and specific data of ATX-a	References
Fish muscle	ATX-a, MCs (-LR, -RR, -YR, -D-3)	LC-MS	15-100 ng/g (ATX-a) 5-100 ng/g (MC-D-3 and -RR) 10-100 ng/g (MC-YR)	LOD: 0.5-7 ng/g LOQ: 1-15 ng/g Recoveries: 70-97%	The method requires only a simple clean-up procedure with SPE-column and no derivatization. For ATX-a: LOD: 7 ng/g, LOQ: 15 ng/g and recoveries: 70-73%	[100]
Water samples	ATX-a, MCs (-LR, -RR, -YR and -D-3)	LC-MS	0.4-30 ng/mL (ATX-a, MC-D-3 and -RR) 0.5-30 ng/mL (MC-LR and -YR)	LOD: 0.2-0.25 ng/mL LOQ: 0.4-0.5 ng/L Recoveries: 84-93%	The method requires only a simple clean-up procedure with SPE-column and no derivatization. For ATX-a: LOD: 0.2 ng/mL, LOQ: 0.4 ng/L and recoveries: 84-85%	[101]
Water samples	ATX-a, STX, MCs (-LR, -YR, -RR and -LA) and NOD	HPLC-ESI-MS/MS	20-1000 ng/L (ATX-a and MCs) 100-10000 ng/L STX	LOD: 27-425 ng/L LOQ: 40-634 ng/L Recoveries: 3.2-96% RSD%: 1.6-5.5%	SPE with RP-C_{18} was used for sample preparation. MCs and NOD have >79% recoveries, ATX-a 50% and STX 3%. For ATX-a: LOD: 30 ng/L, LOQ: 44 ng/L, recovery: 50% and RSD%: 1.9%	[102]

ATX-a: anatoxin-a; ESI: electrospray ionization; HPCL: high-performance liquid chromatography; LC: liquid chromatography; LOD: limit of detection; LOQ: limit of quantification; MC: microcystin; MS: mass spectrometry; MS/MS: tandem mass spectrometry; NOD: nodularin; RP: reversed-phase; RSD: relative standard deviations (Precision, repeatability and reproducibility); SPE: solid-phase extraction; STX: saxitoxin.

Table S3. Risk of bias for the methodological quality of studies reporting different analytical methods for ATX-a determination. 0: not reported; 1: not appropriately or clearly evaluated; 2: appropriately evaluated. M: medium (4-6); L: low (7-8); H: high (0-3).

Reference	Analytical validation (Linear range, LOD, LOQ, RSD, recovery etc.)	Well characterized sample	Full definition of the methodology	Clarity of conclusions	Total	Risk of Bias
			ATX-a alone			
[87]	0	1	1	0	2	H
[104]	0	1	1	0	2	H
[30]	0	1	1	0	2	H
[99]	1	1	2	0	4	M
[98]	1	2	1	1	5	M
[31]	1	2	1	1	5	M
[88]	2	1	2	2	7	L
[89]	0	0	0	1	1	H
[105]	0	2	2	0	4	M
[90]	1	1	1	0	3	H
[91]	1	1	1	0	3	H
[94]	1	1	2	2	6	M
[111]	2	1	2	1	6	M
[108]	0	2	1	0	3	H
[95]	1	2	2	2	7	L
[32]	1	2	2	1	6	M
[33]	0	1	2	0	3	H
[34]	2	2	2	2	8	L
[35]	0	2	2	1	5	M
[36]	1	2	2	2	7	L
[37]	1	2	2	2	7	L
[38]	2	2	2	2	8	L
[39]	2	2	2	2	8	L

[40]	0	2	2	1	5	M
[22]	2	2	2	0	6	M
[41]	2	2	2	2	8	L
[42]	2	2	2	2	8	L
[43]	2	2	2	2	8	L
[44]	1	2	2	1	6	M
[45]	1	1	2	2	6	M
[46]	2	2	2	2	8	L
[47]	2	1	2	2	7	L
[48]	2	2	2	2	8	L
[49]	1	0	2	1	4	M
[50]	2	2	2	2	8	L
[51]	2	2	2	2	8	L
[52]	1	2	2	2	7	L
[53]	2	2	2	0	6	M
[54]	2	2	2	1	7	L
[55]	0	2	2	2	6	M
Cyanotoxins mixtures containing ATX-a						
[100]	2	2	2	0	6	M
[101]	2	2	2	0	6	M
[102]	2	1	2	1	6	M
[56]	2	1	2	2	7	L
[57]	0	2	2	1	5	M
[58]	1	2	2	1	6	M
[59]	1	2	2	1	6	M
[60]	0	2	2	1	5	M
[61]	2	2	2	1	7	L
[62]	1	2	2	2	7	L
[63]	1	1	1	1	4	M
[64]	1	2	2	2	7	L
[65]	2	2	2	2	8	L

[66]	2	2	2	2	8	L
[67]	2	2	2	2	8	L
[68]	2	2	2	2	8	L
[69]	2	1	2	2	7	L
[70]	2	2	2	2	8	L
[71]	2	2	2	2	8	L
[72]	2	2	2	2	8	L
[73]	2	2	2	2	8	L
[74]	2	2	2	2	8	L
[75]	2	2	2	2	8	L
[76]	2	2	2	2	8	L
[77]	2	2	2	2	8	L
[78]	2	2	1	1	6	M
[79]	2	2	2	2	8	L
[80]	2	2	2	2	8	L
[23]	1	2	2	2	7	L
[81]	2	2	2	2	8	L
[82]	0	2	2	2	6	M
[83]	2	2	2	2	7	L
